# Radiomic Model Predicts Lymph Node Response to Induction Chemotherapy in Locally Advanced Head and Neck Cancer

**DOI:** 10.3390/diagnostics11040588

**Published:** 2021-03-25

**Authors:** Michael H. Zhang, David Cao, Daniel T. Ginat

**Affiliations:** 1 Department of Medicine, The University of Chicago, Chicago, IL 60637, USA; Michael.Zhang@uchospitals.edu; 2 Pritzker School of Medicine, The University of Chicago, Chicago, IL 60637, USA; David.Cao@uchospitals.edu; 3 Department of Radiology, The University of Chicago, Chicago, IL 60637, USA

**Keywords:** radiomics, texture analysis, HNSCC, lymph node, CT, induction chemotherapy, cancer, nodal response

## Abstract

This study developed a pretreatment CT-based radiomic model of lymph node response to induction chemotherapy in locally advanced head and neck squamous cell carcinoma (HNSCC) patients. This was a single-center retrospective study of patients with locally advanced HPV+ HNSCC. Forty-one enlarged lymph nodes were found from 27 patients on pretreatment CT and were split into 3:1 training and testing cohorts. Ninety-three radiomic features were extracted. A radiomic model and a combined radiomic-clinical model predicting lymph node response to induction chemotherapy were developed using multivariable logistic regression. Median age was 57 years old, and 93% of patients were male. Post-treatment evaluation was 32 days after treatment, with a median reduction in lymph node volume of 66%. A three-feature radiomic model (minimum, skewness, and low gray level run emphasis) and a combined radiomic-clinical model were developed. The combined model performed the best, with AUC = 0.85 on the training cohort and AUC = 0.75 on the testing cohort. A pretreatment CT-based lymph node radiomic signature combined with clinical parameters was able to predict nodal response to induction chemotherapy for patients with locally advanced HNSCC.

## 1. Introduction

Head and neck squamous cell carcinoma (HNSCC) is a common malignancy with more than 800,000 new cases diagnosed every year globally, the majority of them presenting with locally advanced disease at the time of diagnosis [1]. Patients with locally advanced disease are typically treated with a combination of surgery, radiation, and chemotherapy, but response to clinical treatment varies immensely among patients and outcomes have remained relatively stagnant and unsatisfactory over the past decade [1,2,3]. In HNSCC, nodal disease remains the most important marker of prognosis; the presence of lymph node metastasis is the most accurate predictor of cancer-related outcomes, and extracapsular spread in metastatic lymph nodes is associated with another drop in overall survival [4,5,6]. A few other prognostic biomarkers have been validated, namely, human papillomavirus (HPV) status, PD-L1 status, and 18F-fluorodeoxyglucose (FDG) uptake on PET imaging [7,8,9,10]. Because of the remarkably heterogenous responses to treatment, there is a need for better prognostic biomarkers to identify those who will and will not respond to treatment and to further personalize treatment regimens.

Radiomics, the field of quantifying image intensity, shape, and textural characteristics through the use of high-throughput data-characterization algorithms, has been proposed as a non-invasive and accessible method to analyze tumors. Radiomic models have previously been studied in a multitude of tumors, across virtually every imaging modality [11,12,13,14]. In the realm of HNSCC, radiomics has been utilized to differentiate malignant from benign tissue, to assess HPV status, and to identify underlying driver mutations [15,16,17]. Other studies have used radiomic signatures as prognostic biomarkers and even to predict side effects of treatment such as xerostomia or weight loss [18,19,20,21].

There is a need for better predictors of response to HNSCC treatment. Given the importance of nodal disease to the overall prognosis of HNSCC, this study aims to develop a CT-based radiomic biomarker to predict lymph node response to induction chemotherapy in patients with locally advanced HNSCC [22].

## 2. Materials and Methods

### 2.1. Patient Population and Treatment

This retrospective study was approved by the institutional review board at the University of Chicago, and informed consent was waived. A retrospective review of patients with pathology-proven stage IVa or IVb HNSCC, enrolled in a response-adapted volume de-escalation trial between May 2010 and March 2014 at our institution, was included in this study [23]. Patients were 18 years of age or older with Karnofsky performance status of ≥70% and normal organ and marrow function. Patients were excluded if they had HPV-negative HNSCC, if they did not have pretreatment contrast-enhanced CT imaging available for radiomic analysis, or if no enlarged lymph nodes were seen on pretreatment imaging (defined as short axis diameter >15 mm). Patients with prior radiotherapy or chemotherapy, symptomatic peripheral neuropathy, current immunosuppressive therapy, or metastatic disease were excluded. Ultimately, 41 lymph nodes from 27 patients were included.

All patients underwent two 21-day cycles of an induction chemotherapy regimen of cisplatin, paclitaxel, and escalating doses of cetuximab and everolimus. Post-treatment cross-sectional evaluation of individual lymph node response was performed approximately 1 month after the induction regimen (median time interval 31 days, range 29–38 days). Volume response was measured as a percent change between the pretreatment and post-treatment lymph node volume.

### 2.2. CT Image Acquisition

Head and neck CT scans were acquired after intravenous injection of typically 50 to 65 mL of nonionic iodinated contrast medium (350 mg of iodine per milliliter, Omnipaque) at a rate of 1.2 mL/s and 55 second delay after the start of the injection. The scan parameters included 120 kV; 250 mAs; rotation time, 1.0 second; pitch, 0.75; collimation, 24 × 1.2 mm^2^ with a B30s smoothing algorithm, section thicknesses of 3 mm, and display field of view of 20 to 25 cm.

### 2.3. CT Texture Analysis

**Figure 1:**
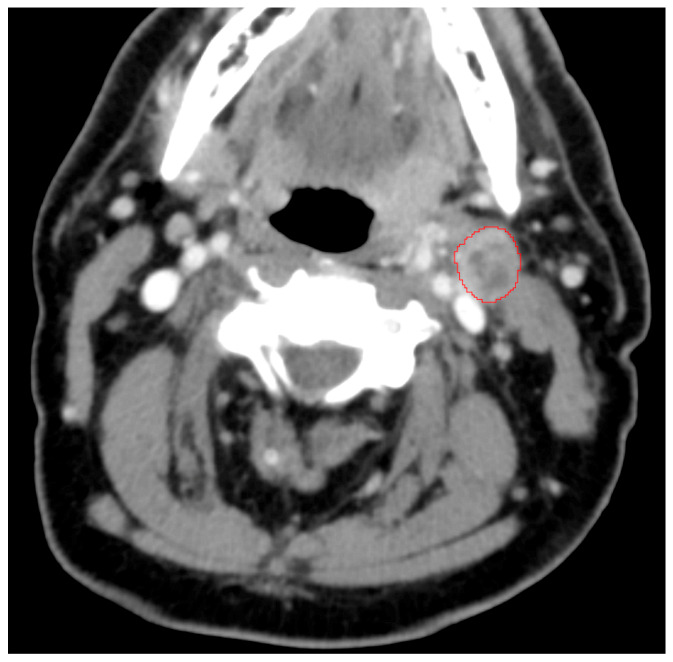
An enlarged lymph node and region of interest outlined on a pretreatment axial CT image.

Pretreatment CT images were analyzed using 3D Slicer 4.10.2 [24]. The axial slice with the largest lymph node cross-sectional area assessed by visual estimation was selected for segmentation. Region of interest (ROI) segmentation was manually performed for each identified lymph node under the supervision of a board-certified neuroradiologist (Figure 1). Ninety-three texture features from 6 classes—first-order features, gray level co-occurrence matrix (GLCM) features, gray level size zone matrix (GLSZM) features, gray level run length matrix (GLRLM) features, neighboring gray tone difference matrix (NGTDM) features, and gray level dependence matrix (GLDM) features—were calculated from each ROI using the Slicer pyradiomics module, which is compliant with the Imaging Biomarker Standardisation Initiative [25,26]. These 6 classes and 93 features are described in Supplemental S1.

### 2.4. Radiomic Feature Selection

A radiomic model was developed to logistically predict how lymph nodes would respond to induction chemotherapy. The median reduction in lymph node volume of 66% was used as the cutoff between good response (GR) and poor response (PR) (Figure S1). GR was defined as >66% reduction in the target lymph node. Patients with ≤66% decrease in the sum of target lesions were classified as PR.

We started by splitting the dataset of 41 lymph nodes into a training cohort (*n* = 30) and testing cohort (*n* = 11). The feature selection process and radiomic model development were done exclusively on the training cohort. All 93 features were linearly regressed against the percent reduction in lymph node volume. Features with *p* ≥ 0.05 after adjustment by the false discovery rate (FDR) were considered statistically insignificant and removed. Next, the radiomic features were assessed for collinearity to avoid redundancy in the model. Any feature that was highly correlated with another feature, defined as a Pearson correlation coefficient |R^2^| > 0.8, was removed. Lastly, a least absolute shrinkage and selection operator (LASSO) regression was applied to identify the most useful and prognostic features while simultaneously eliminating nonpredictive features. The optimal LASSO regularization parameter λ was determined by a 5-fold cross-validation. After the feature selection process, we were left with three radiomic features: minimum, skewness, and low gray level run emphasis (LGRE).

### 2.5. Radiomic Model Construction and Evaluation

A multivariable logistic regression model was performed on the training cohort. This model was then evaluated on the testing cohort. A clinical model (patient age, sex, and TMN staging) was developed on the training cohort for comparison. Lastly, a model combining the radiomic model and clinical features was developed. In the combined model, all the clinical parameters in addition to a predictive Rad-score (which ranged from 0 to 1 with a greater Rad-score predicting higher likelihood of GR) was used. Associated receiver operating characteristic (ROC) curves were plotted for all three models. All statistical analyses were performed with R, Version 4.0.3 (www.r-project.org, accessed on 12 October 2020, Vienna Austria).

## 3. Results

### 3.1. Demographic and Clinical Characteristics

We retrospectively analyzed 41 lymph nodes from 27 patients with locally advanced HNSCC who were treated between 2010 and 2014 (Table 1). The median patient age of our study population was 57 years old, and 93% were male. At a median follow-up of 32 days, the median reduction in lymph node volume was 66%. There were no significant differences in the clinical characteristics of our training and testing populations besides the T stage. Overall stage and N stage were not significantly different between our cohorts.

### 3.2. Radiomic Feature Selection

We extracted 93 radiomic features from pretreatment contrast-enhanced CT images (Supplemental S1). Each feature was linearly regressed against the percent change in lymph node volume. Of the 93 features, 65 had a statistically significant regression with FDR-adjusted *p*-value < 0.05 (Table S1 Supplementary Materials). Next, these 65 features were examined for collinearity. Highly colinear variables were removed, and 14 radiomic features remained. A LASSO regression was then performed to eliminate nonsignificant features (Figure S1). A logistic regression model using two first-order features (minimum, skewness) and one gray level run length matrix feature (LGRE) was produced (Table 2). Descriptions of these features can be found in Supplemental S1. Minimum pixel intensity and low gray level run emphasis were positively associated with good lymph node response, while skewness was associated with poorer lymph node response.

### 3.3. Evaluation of Radiomic and Clinical Models

**Figure 2:**
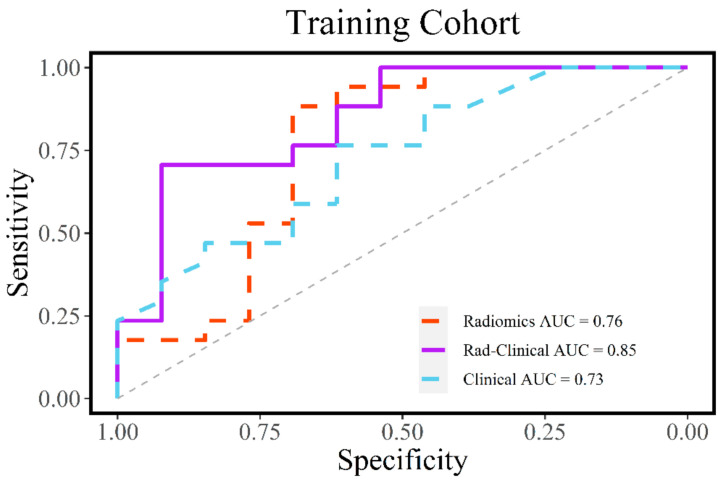
Receiver operating characteristic (ROC) curve analysis for the radiomic, clinical, and combined models in the training cohort. The combined model performed the best.

**Figure 3:**
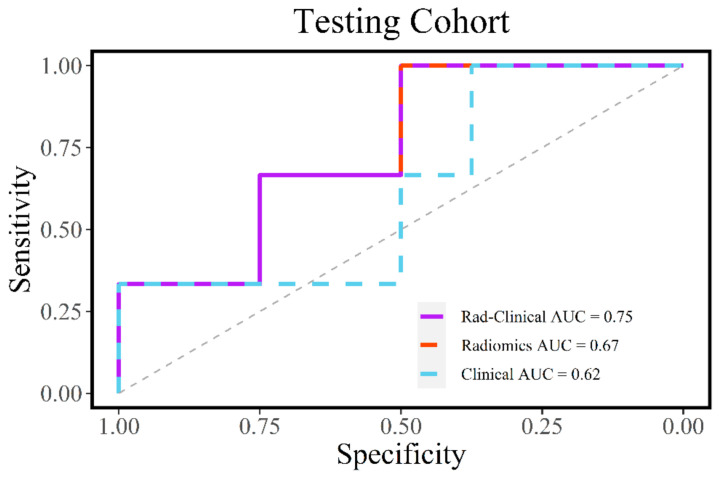
Receiver operating characteristic (ROC) curve analysis for the radiomic, clinical, and combined models in the testing cohort. The combined model performed the best.

The radiomic model was compared with a clinical model (comprised of age, sex, T stage, and N stage) and a combined radiomic-clinical model (comprised of Rad-score, age, sex, T stage, and N stage). All three models were developed on the training cohort and then validated on the testing cohort. On the training cohort, the combined model did the best (AUC = 0.85), followed by the radiomic (AUC = 0.76) and then clinical model (AUC = 0.73) (Figure 2). In the testing cohort, the combined model (AUC = 0.75) outperformed the radiomic (AUC = 0.67) and clinical models (AUC = 0.62) (Figure 3). The combined model was 71% sensitive and 92% specific in our training cohort (Table 3). In our testing cohort, the model was 100% sensitive and 50% specific (Table 4).

## 4. Discussion

Recent advances in the field of radiomics have allowed for the extraction of informative imaging features to help quantify differences in tumors as seen on imaging. CT-based radiomic features have been used to predict HPV status and extranodal metastasis [27,28,29]. Radiomic signatures have been used to evaluate intratumoral heterogeneity, something that is difficult to do by tissue biopsy, and were found to be associated with different gene-expression patterns [30,31]. However, there has been minimal work done evaluating individual lymph nodes prior to oncologic treatment. The eighth edition of the pathological tumor-node-metastasis staging classification uses the number, size, and laterality of metastatic lymph nodes and incorporates two new parameters: depth of invasion and extranodal extension [32,33]. Other approaches have used the lymph node ratio (defined as the proportion of metastatic lymph nodes related to all examined nodes) to predict prognosis [34]. León et al. included weighted lymph node ratios to further adjust prognosis for the presence of each node with extracapsular spread [35]. There appears to be prognostic value in the attributes of each individual lymph node. This study is, to our knowledge, the first to develop a radiomic-based model to predict lymph node response after induction chemotherapy.

In this study, we extracted and analyzed 93 radiomic features characterizing enlarged lymph nodes in patients with HNSCC. We developed a logistic regression classifier using three extracted radiomic features. The features utilized in our radiomic model include two first-order features (minimum and skewness) as well as LGRE. Lower minimum voxel intensity predicted poor nodal response to treatment. The minimum attenuation was −5 Hounsfield units (HU) among PR nodes and 18 HU among GR nodes, likely characterizing the low attenuating nature of necrotic lymph nodes. Likewise, LGRE measures the concentration of low attenuating voxels in the ROI. A higher LGRE value (representing a greater concentration of low gray-level values) predicted worse nodal response. Skewness has been used to characterize intratumoral heterogeneity [36,37,38]. Chen et al. used skewness and minimum in a four-feature signature to classify lung nodules as benign or malignant on CT imaging [39]. It has been previously hypothesized that tumor heterogeneity manifests at different spatial scales, from the macroscopic down to the cellular and genetic level [31,40,41]. It is unsurprising that the features elicited in our model to predict lymph node response represent radiologic heterogeneity.

Our radiomic model was able to predict a GR to induction chemotherapy with an AUC of 0.76 in the training cohort. In the testing cohort, the radiomic model had some predictive ability though this was diminished from the training cohort. Notably, the integration of our radiomic model as a Rad-score with previously available clinical information improved predictive ability in both our training (AUC = 0.85) and testing cohorts (AUC = 0.75). This is in line with previous studies which have found similar synergistic improvements between radiomic and clinical-based predictions [20,42]. Recently, Zhai et al. demonstrated and externally validated a combined radiomic-clinical model to predict individual lymph node failure after definitive radiotherapy with a c-index = 0.80 in the internal validation cohort and c-index = 0.71 in the externally validated study [42,43]. Like our model, their model used a combined radiomic and clinical approach, signifying that there is useful and complementary information that can be extracted from CT imaging of lymph nodes. However, their model requires 3D segmentation of each lymph node, which is significantly more time-consuming than using only one axial slice for feature extraction. 

Our study demonstrates that radiomic features of lymph nodes on pretreatment CT imaging can provide useful information in predicting the response to induction chemotherapy. Ideally, prediction of individual nodal response to induction chemotherapy could lead to more personalized radiation dose intensification focusing on high-risk nodes or even direct surgical dissection for high-risk nodes. However, this is only a preliminary study. While we were able to internally validate our model, external multi-center validation is the next step.

## 5. Conclusions

A pretreatment CT-based lymph node radiomic signature combined with clinical parameters is able to predict nodal response to induction chemotherapy for patients with locally advanced HNSCC. Future studies are needed to externally validate this model.

## Figures and Tables

**Table 1:** Clinical characteristics of the patient population.

	All Subjects	Training Cohort	Testing Cohort	*p*-Value
*n*	41	30	11	
Age (years)	57 ± 6	58 ± 7	56 ± 3	0.57
Sex				
Female	3	3	0	0.68
Male	38	27	11	
Time Interval (days)	32 ± 3	32 ± 3	33 ± 3	0.48
Lymph Node Reduction (%)	66% [53–82%]	77% [53–82%]	62% [58–67%]	0.34
Lymph Node Response				0.19
Good Response (> 66%)	20	17	3	
Poor Response (≤ 66%)	21	13	8	
Overall Stage				0.95
IVa	39	28	11	
IVb	2	2	0	
T Stage				0.04
1	6	5	1	
2	15	7	8	
3	11	10	1	
4	9	8	1	
N Stage				0.75
2a	2	1	1	
2b	20	14	6	
2c	18	14	4	
3	1	1	0	

Time interval is the number of days between the pretreatment scan and follow-up scan. Change in lymph node volume was assessed at this time. Lymph node reduction is the percent change in volume of the lymph node between the pretreatment and follow-up scan. Numerical data are mean ± standard deviation or median [interquartile range].

**Table 2:** Logistic regression model of radiomic features to predict good lymph node response.

	Correlation Coefficient	Beta Coefficient ± SE	*p* Value
(Intercept)		−1.26 ± 1.09	0.25
Minimum	0.0045	0.014 ± 0.015	0.35
Skewness	−0.083	−0.49 ± 0.39	0.21
Low Gray Level Run Emphasis	1.20	9.89 ± 7.11	0.16

Good response is >66% and poor response is ≤66% reduction in lymph node volume. Correlation coefficient is the relationship between feature value and percent reduction in lymph node volume. Positive value indicates increase in feature value is correlated with greater percent reduction in lymph node volume. SE = standard error.

**Table 3:** Confusion matrix showing the combined radiomic-clinical model performance in the training cohort.

	Predicted: Good Response	Predicted: Poor Response	
Observed: good response	12	5	17
Observed: poor response	1	12	13
	13	17	

*n* = 30. Positive predictive value 92%. Negative predictive value 71%. Sensitivity 71%. Specificity 92%.

**Table 4:** Confusion matrix showing the combined radiomic-clinical model performance in the testing cohort.

	Predicted: Good Response	Predicted: Poor Response	
Observed: good response	3	0	3
Observed: poor response	4	4	8
	7	4	

*n* = 11. Positive predictive value 43%. Negative predictive value 100%. Sensitivity 100%. Specificity 50%.

## Data Availability

The data presented in this study are available on request from the corresponding author.

## Supplementary Materials

The following are available online at https://www.mdpi.com/2075-4418/11/4/588/s1, Supplemental S1: radiomic features and descriptions, Table S1: linear regression, Figure S1: LASSO regression optimization.
