# Supplementary material for: Radiomic Model Predicts Lymph Node Response to Induction Chemotherapy in Locally Advanced Head and Neck Cancer
Source: Diagnostics (Basel). 2021 Mar 25;11(4):588. doi: 10.3390/diagnostics11040588 (PMC8064478; doi:10.3390/diagnostics11040588)
Supplement: Supplementary file 1 [file diagnostics-11-00588-s001.pdf]

Supplemental 1: Descriptions and algorithms for extracted radiomic features. In this study, a total of 93 radiomic features were extracted from each region of interest (ROI) in Python (pyradiomics; version: 3.0). These features were divided into three types: first-order statistics, gray level cooccurrence matrix, gray level run length matrix, gray level size zone matrix, neighbouring gray tone difference matrix and gray level dependence matrix. The following algorithms and descriptions are from pyradiomics (https://pyradiomics.readthedocs.io/en/latest/).

First-order features

First-order statistics describe the distribution of voxel intensity values within the ROI through commonly used and basic metrics.

Let:

X be a set of N_P voxels included in the ROI,

$P(i)$ be the first order histogram with N_g discrete intensity levels, where N_g is the number of non-zero bins, equally spaced from 0 with a width defined in the binWidth parameter,

$p(i)$ be the normalized first order histogram and equal to $\frac{P(i)}{N_P}$.

1. Energy:

$$energy = \sum_{i=1}^{N_P} (X(i) + c)^2$$

2. Total Energy

$$total\ energy = V_{voxel} \sum_{i=1}^{N_P} (X(i) + c)^2$$

3. Entropy:

$$entropy = -\sum_{i=1}^{N_g} p(i) \log_2(p(i) + \epsilon)$$

Here, ϵ is an arbitrarily small positive number ($\approx 2.2\times10^{-16}$)

4. Minimum:

$$minimum = \min(X)$$

5. 10th Percentile

The 10th percentile of X.

6. 90th Percentile

The 90th percentile of X.

7. Maximum:

The maximum gray level intensity within the ROI.

$$maximum = \max(X)$$

8. Mean:

The average gray level intensity within the ROI.

$$mean = \frac{1}{N_P} \sum_{i=1}^{N_P} X(i)$$

9. Median:
The median gray level intensity within the ROI.

10. Interquartile Range:

$$interquartile\ range = P_{75} - P_{25}$$

Here P_{25} and P_{75} are the 25th and 75th percentile of the image array, respectively.

11. Range:

$$range = \max(X) - \min(X)$$

12. Mean Absolute Deviation (MAD):
MAD is the mean distance of all intensity values from the Mean Value of the ROI.

$$MAD = \frac{1}{N_P} \sum_{i=1}^{N_P} |X(i) - \bar{X}|$$

Where $\bar{X}$ is the mean of X

13. Robust Mean Absolute Deviation (rMAD):
Robust Mean Absolute Deviation is the mean distance of all intensity values from the Mean Value calculated on the subset of image array with gray levels in between, or equal to the 10th and 90th percentile.

$$rMAD = \frac{1}{N_{10-90}} \sum_{i=1}^{N_{10-90}} |X_{10-90}(i) - \bar{X}_{10-90}|$$

14. Root Mean Square (RMS):

$$RMS = \sqrt{\frac{\sum_{i=1}^{N_P} (X(i) + c)^2}{N_P}}$$

Here, c is optional value, defined by *voxelArrayShift*, which shifts the intensities to prevent negative values in X. This ensures that voxels with the lowest gray values contribute the least to RMS, instead of voxels with gray level intensity closest to 0.

15. Skewness:
Skewness measures the asymmetry of the distribution of values about the Mean value. Depending on where the tail is elongated and the mass of the distribution is concentrated, this value can be positive or negative.

$$\boldsymbol{skewness} = \frac{\frac{\mathbf{1}}{\boldsymbol{N_P}} \sum_{\boldsymbol{i}=\mathbf{1}}^{\boldsymbol{N_P N}} (\boldsymbol{X}(\boldsymbol{i}) - \bar{\boldsymbol{X}})^{\mathbf{3}}}{\left(\sqrt{\frac{\mathbf{1}}{\boldsymbol{N_P}} \sum_{\boldsymbol{i}=\mathbf{1}}^{\boldsymbol{N_P}} (\boldsymbol{X}(\boldsymbol{i}) - \bar{\boldsymbol{X}})^{\mathbf{2}}}\right)^{\mathbf{3}}}$$

where $\bar{\boldsymbol{X}}$ is the mean of $\boldsymbol{X}$.

16. Kurtosis:

$$\boldsymbol{kurtosis} = \frac{\frac{1}{N_P}\sum_{i=1}^{N_P}(X(i)-\bar{X})^4}{\left(\sqrt{\frac{1}{N_P}\sum_{i=1}^{N_P}(X(i)-\bar{X})^2}\right)^2}$$

Where $\bar{X}$ is the mean of X.
Kurtosis is a measure of the “peakedness” of the distribution of values in the image ROI. A higher kurtosis implies that the mass of the distribution is concentrated towards the tail(s) rather than towards the mean. A lower kurtosis implies the reverse: that the mass of the distribution is concentrated towards a spike near the Mean value.

17. Variance:

$$\boldsymbol{variance} = \frac{1}{N_P}\sum_{i=1}^{N_P}(X(i)-\bar{X})^2$$

18. Uniformity:

$$\boldsymbol{uniformity} = \sum_{i=1}^{N_g} P(i)^2$$

Gray Level Co-occurrence Matrix (GLCM) Features

A normalized GLCM is defined as $P(i,j;\delta,\alpha)$, a matrix with size $N_g \times N_g$ describing the second-order joint probability function of an image, where the $(i,j)^{\mathrm{th}}$ element represents the number of times the combination of intensity levels i and j occur in two pixels in the image, that are separated by a distance of δ pixels in direction α. The distance δ from the center voxel is defined as the distance according to the infinity norm. For δ=1, this results in 2 neighbors for each of 13 angles in 3D (26-connectivity).

Let:

ϵ be an arbitrarily small positive number ($\approx 2.2\times10^{-16}$)

$P(i,j)$ be the co-occurence matrix for an arbitrary δ and α, ,

$p(i,j)$ be the normalized co-occurence matrix and equal to $\frac{P(i,j)}{\sum P(i,j)}$,

N_g be the number of discrete intensity levels in the image,

$p_x(i) = \sum_{j=1}^{N_g} P(i,j)$ be the marginal row probabilities,

$p_y(i) = \sum_{i=1}^{N_g} P(i,j)$ be the marginal column probabilities,

μ_x be the mean gray level intensity of p_x,

μ_y be the mean gray level intensity of p_y,

σ_x be the standard deviation of p_x,

σ_y be the standard deviation of p_y,

$p_{x+y}(k) = \sum_{i=1}^{N_g}\sum_{j=1}^{N_g} P(i,j)$, where $i+j=k$, and $k = 2,3,\ldots,2N_g$,

$p_{x-y}(k) = \sum_{i=1}^{N_g}\sum_{j=1}^{N_g} P(i,j)$, where $|i-j| = k$, and $k = 0,1,\ldots,N_g-1$,

$HX = -\sum_{i=1}^{N_g} p_x(i)\log_2[p_x(i)+\epsilon]$ be the entropy of p_x,

$HY = -\sum_{i=1}^{N_g} p_y(i)\log_2[p_y(i)+\epsilon]$ be the entropy of p_y,

$HXY = -\sum_{i=1}^{N_g}\sum_{j=1}^{N_g} P(i,j)\log_2[P(i,j)+\epsilon]$ be the entropy of $P(i,j)$,

$HXY1 = -\sum_{i=1}^{N_g}\sum_{j=1}^{N_g} P(i,j)\log_2(p_x(i)p_y(j)+\epsilon)$,

$$HXY2 = -\sum_{i=1}^{N_g}\sum_{j=1}^{N_g} p_x(i)p_y(j)\log_2(p_x(i)p_y(j) + \epsilon)$$

1. Autocorrelation
Autocorrelation is a measure of the magnitude of the fineness and coarseness of texture.

$$autocorrelation = \sum_{i=1}^{N_g}\sum_{j=1}^{N_g} ijp(i,j)$$

2. Joint Average

$$joint\ average = \mu x = \sum_{i=1}^{N_g}\sum_{j=1}^{N_g} p(i,j)i$$

3. Cluster Prominence:

$$cluster\ prominence = \sum_{i=1}^{N_g}\sum_{j=1}^{N_g} (i + j - \mu_x - \mu_y)^4 p(i,j)$$

4. Cluster Shade:

$$cluster\ shade = \sum_{i=1}^{N_g}\sum_{j=1}^{N_g} (i + j - \mu_x - \mu_y)^3 p(i,j)$$

5. Cluster Tendency:

$$cluster\ tendency = \sum_{i=1}^{N_g}\sum_{j=1}^{N_g} (i + j - \mu_x - \mu_y)^2 p(i,j)$$

6. Contrast:

$$contrast = \sum_{i=1}^{N_g}\sum_{j=1}^{N_g} (i - j)^2 p(i,j)$$

Contrast is a measure of the local intensity variation, favoring values away from the diagonal ($i = j$). A larger value correlates with a greater disparity in intensity values among neighboring voxels.

7. Correlation:

$$correlation = \frac{\sum_{i=1}^{N_g}\sum_{j=1}^{N_g} ijp(i,j) - \mu_x\mu_y}{\sigma_x(i)\sigma_y(j)}$$

8. Difference Average:

$$difference\ average = \sum_{k=0}^{N_g-1} kp_{x-y}(k)$$

9. Difference Entropy:

$$\boldsymbol{difference\ entropy} = \sum_{k=0}^{N_g-1} p_{x-y}(k)\log_2(p_{x-y}(k)+\epsilon)$$

10. Difference Variance:

$$\boldsymbol{difference\ variance} = \sum_{k=0}^{N_g-1} (k-DA)^2 p_{x-y}(k)$$

11. Joint Energy:

$$\boldsymbol{joint\ energy} = \sum_{i=1}^{N_g}\sum_{j=1}^{N_g} (p(i,j))^2$$

12. Joint Entropy:

$$\boldsymbol{joint\ entropy} = -\sum_{i=1}^{N_g}\sum_{j=1}^{N_g} p(i,j)\log_2(p(i,j)+\epsilon)$$

13. Informal Measure of Correlation (IMC) 1:

$$IMC1 = \frac{HXY - HXY1}{\max\{HX, HY\}}$$

14. Informal Measure of Correlation (IMC) 2:

$$IMC2 = \sqrt{1 - e^{-2(HXY2 - HXY)}}$$

15. Inverse Difference Moment (IDM)

$$IDM = \sum_{i=1}^{N_g}\sum_{j=1}^{N_g} \frac{p(i,j)}{1+|i-j|^2}$$

16. Maximal Correlation Coefficient (MCC)

$$MCC = \sqrt{second\ largest\ eigenvalue\ of\ Q}$$

$$Q(i,j) = \sum_{k=0}^{N_g} \frac{p(i,k)p(j,k)}{p_x(i)p_y(k)}$$

17. Inverse Difference Moment Normalized (IDMN):

$$IDMN = \sum_{i=1}^{N_g}\sum_{j=1}^{N_g} \frac{p(i,j)}{1+\left(\frac{|i-j|^2}{{N_g}^2}\right)}$$

18. Inverse Difference (ID):

$$ID = \sum_{i=1}^{N_g}\sum_{j=1}^{N_g} \frac{p(i,j)}{1+|i-j|}$$

19. Inverse Difference Normalized (IDN):

$$\boldsymbol{IDN} = \sum_{i=1}^{N_g}\sum_{j=1}^{N_g}\frac{\boldsymbol{p}(\boldsymbol{i},\boldsymbol{j})}{\mathbf{1} + \left(\frac{|\boldsymbol{i}-\boldsymbol{j}|}{\boldsymbol{N_g}}\right)}$$

20. Inverse Variance:

$$\boldsymbol{inverse\ variance} = \sum_{i=1}^{N_g}\sum_{j=1}^{N_g}\frac{\boldsymbol{p}(\boldsymbol{i},\boldsymbol{j})}{|\boldsymbol{i}-\boldsymbol{j}|^2} \quad ,\boldsymbol{i} \neq \boldsymbol{j}$$

21. Maximum Probability:

$$\boldsymbol{maximum\ probability} = \max(\boldsymbol{p}(\boldsymbol{i},\boldsymbol{j}))$$

22. Sum Average:

$$\boldsymbol{sum\ average} = \sum_{k=2}^{2N_g}(\boldsymbol{k}\boldsymbol{p}_{\boldsymbol{x}+\boldsymbol{y}}(\boldsymbol{k}))$$

23. Sum Entropy:

$$\boldsymbol{sum\ entropy} = -\sum_{k=2}^{2N_g}\boldsymbol{p}_{\boldsymbol{x}+\boldsymbol{y}}(\boldsymbol{k})\,\mathbf{log}_2(\boldsymbol{p}_{\boldsymbol{x}+\boldsymbol{y}}(\boldsymbol{k}) + \boldsymbol{\epsilon})$$

24. Sum of Squares:

$$\boldsymbol{sum\ squares} = \sum_{i=1}^{N_g}\sum_{j=1}^{N_g}(\boldsymbol{i}-\boldsymbol{\mu_x})^2\boldsymbol{p}(\boldsymbol{i},\boldsymbol{j})$$

Gray Level Size Zone Matrix (GLSZM) Features

A GLSZM describes the amount of homogeneous connected areas within the volume, of a certain size and intensity, thereby describing heterogeneity in the image at a regional scale. A gray level zone is defined as the number of connected voxels that share the same gray level intensity. A voxel is considered connected if the distance is 1 according to the infinity norm (26-connected region in 3D). In a GLSZM $\boldsymbol{P}(\boldsymbol{i},\boldsymbol{j})$, the $(\boldsymbol{i},\boldsymbol{j})^{\mathbf{th}}$element equals the number of zones with gray level $\boldsymbol{i}$ and size $\boldsymbol{j}$ appear in image. Contrary to GLCM and GLRLM, the GLSZM is rotation independent, with only one matrix calculated for all directions in the ROI. The mathematical formulas that define the GLSZM features correspond to the definitions of features extracted from the GLRLM.

Let:

$\boldsymbol{N_g}$ be the number of discreet intensity values in the image,

$\boldsymbol{N_s}$ be the number of discreet zone sizes in the image,

$\boldsymbol{N_p}$ be the number of voxels in the image,

$\boldsymbol{N_z}$ be the number of zones in the ROI, which is equal to $\sum_{i=1}^{N_g}\sum_{j=1}^{N_s}\boldsymbol{P}(\boldsymbol{i},\boldsymbol{j})$, and $\mathbf{1} \leq \boldsymbol{N_z}\boldsymbol{N_z} \leq \boldsymbol{N_p}$

$\boldsymbol{P}(\boldsymbol{i},\boldsymbol{j})$ be the size zone matrix,

$\boldsymbol{p}(\boldsymbol{i},\boldsymbol{j})$ be the normalized size zone matrix, defined as $\boldsymbol{p}(\boldsymbol{i},\boldsymbol{j}) = \frac{\boldsymbol{P}(\boldsymbol{i},\boldsymbol{j})}{\boldsymbol{N_z}}$.

1. Small Area Emphasis (SAE):

$$SAE = \frac{\sum_{i=1}^{N_g} \sum_{j=1}^{N_s} \frac{P(i,j)}{j^2}}{N_z}$$

2. Large Area Emphasis (LAE):

$$LAE = \frac{\sum_{i=1}^{N_g} \sum_{j=1}^{N_s} P(i,j) j^2}{N_z}$$

3. Gray Level Non-Uniformity (GLN):

$$GLN = \frac{\sum_{i=1}^{N_g} \left(\sum_{j=1}^{N_s} P(i,j) \right)^2}{N_z}$$

GLN measures the variability of gray-level intensity values in the image, with a lower value indicating more homogeneity in intensity values.

4. Gray Level Non-Uniformity Normalized (GLNN):

$$GLNN = \frac{\sum_{i=1}^{N_g} \left(\sum_{j=1}^{N_s} P(i,j) \right)^2}{{N_z}^2}$$

GLNN measures the variability of gray-level intensity values in the image, with a lower value indicating a greater similarity in intensity values. This is the normalized version of the GLN formula.

5. Size-Zone Non-Uniformity (SZN):

$$SZN = \frac{\sum_{j=1}^{N_s} \left(\sum_{i=1}^{N_g} P(i,j) \right)^2}{N_z}$$

6. Size-Zone Non-Uniformity Normalized (SZNN):

$$SZNN = \frac{\sum_{j=1}^{N_s} \left(\sum_{i=1}^{N_g} P(i,j) \right)^2}{{N_z}^2}$$

7. Zone Percentage (ZP):

$$ZP = \frac{N_z}{N_p}$$

8. Gray Level Variance (GLV):

$$GLV = \sum_{i=1}^{N_g} \sum_{j=1}^{N_s} p(i,j)\,(i - \mu)^2$$

Here, $\mu = \sum_{i=1}^{N_g} \sum_{j=1}^{N_s} p(i,j)\, i$

9. Zone Variance (ZV):

$$ZV = \sum_{i=1}^{N_g} \sum_{j=1}^{N_s} p(i,j)\,(j - \mu)^2$$

Here, $\mu = \sum_{i=1}^{N_g} \sum_{j=1}^{N_s} p(i,j)\, j$

10. Zone Entropy (ZE):

$$\boldsymbol{ZE} = \sum_{\boldsymbol{i}=1}^{\boldsymbol{N_g}} \sum_{\boldsymbol{j}=1}^{\boldsymbol{N_s}} \boldsymbol{p}(\boldsymbol{i},\boldsymbol{j}) \log_2[\boldsymbol{p}(\boldsymbol{i},\boldsymbol{j}) + \boldsymbol{\epsilon}]$$

Here, $\boldsymbol{\epsilon}$ is an arbitrarily small positive number ($\approx 2.2 \times 10^{-16}$)

11. Low Gray Level Zone Emphasis (LGLZE):

$$\boldsymbol{LGLZE} = \frac{\sum_{i=1}^{N_g} \sum_{j=1}^{N_s} \frac{P(i,j)}{i^2}}{N_z}$$

12. High Gray Level Zone Emphasis (HGLZE):

$$\boldsymbol{HGLZE} = \frac{\sum_{i=1}^{N_g} \sum_{j=1}^{N_s} i^2 P(i,j)}{N_z}$$

13. Small Area Low Gray Level Emphasis (SALGLE):

$$\boldsymbol{SALGLE} = \frac{\sum_{i=1}^{N_g} \sum_{j=1}^{N_s} \frac{P(i,j)}{i^2 j^2}}{N_z}$$

14. Small Area High Gray Level Emphasis (SAHGLE):

$$\boldsymbol{SAHGLE} = \frac{\sum_{i=1}^{N_g} \sum_{j=1}^{N_s} \frac{P(i,j) i^2}{j^2}}{N_z}$$

15. Large Area Low Gray Level Emphasis (LALGLE):

$$\boldsymbol{LALGLE} = \frac{\sum_{i=1}^{N_g} \sum_{j=1}^{N_s} \frac{P(i,j) j^2}{i^2}}{N_z}$$

16. Large Area High Gray Level Emphasis (LAHGLE):

$$\boldsymbol{HGLZE} = \frac{\sum_{i=1}^{N_g} \sum_{j=1}^{N_s} i^2 j^2 P(i,j)}{N_z}$$

Gray Level Run Length Matrix (GLRLM) Features

A GLRLM quantifies gray level runs, which are defined as the length in number of pixels, of consecutive pixels that have the same gray level value. In a gray level run length matrix $\boldsymbol{P}(\boldsymbol{i},\boldsymbol{j}|\boldsymbol{\theta})$, the $(\boldsymbol{i},\boldsymbol{j})^{\text{th}}$element describes the number of runs with gray level $\boldsymbol{i}$ and length $\boldsymbol{j}$ occur in the image along angle $\boldsymbol{\theta}$.

Let:

$\boldsymbol{N_g}$ be the number of discreet intensity values in the image,

$\boldsymbol{N_r}$ be the number of discreet run lengths in the image,

$\boldsymbol{N_p}$ be the number of voxels in the image,

$\boldsymbol{N_z}(\boldsymbol{\theta})$ be the number of runs in the image along angle $\boldsymbol{\theta}$, which is equal to $\sum_{i=1}^{N_g} \sum_{j=1}^{N_r} \boldsymbol{P}(\boldsymbol{i},\boldsymbol{j}|\boldsymbol{\theta})$, and $\boldsymbol{1} \le \boldsymbol{N_z}(\boldsymbol{\theta}) \le \boldsymbol{N_p}$,

$\boldsymbol{P}(\boldsymbol{i},\boldsymbol{j}|\boldsymbol{\theta})$ be the run length matrix for an arbitrary direction $\boldsymbol{\theta}$,

$\boldsymbol{p}(\boldsymbol{i},\boldsymbol{j}|\boldsymbol{\theta})$ be the normalized run length matrix, defined as $\boldsymbol{p}(\boldsymbol{i},\boldsymbol{j}|\boldsymbol{\theta}) = \frac{P(i,j|\theta)}{N_z(\theta)}$,

1. Short Run Emphasis (SRE):

$$\boldsymbol{SRE} = \frac{\sum_{i=1}^{N_g}\sum_{j=1}^{N_r}\frac{P(i,j|\theta)}{j^2}}{N_z(\theta)}$$

2. Long Run Emphasis (LRE):

$$\boldsymbol{LRE} = \frac{\sum_{i=1}^{N_g}\sum_{j=1}^{N_r} j^2 P(i,j|\theta)}{N_z(\theta)}$$

3. Gray Level Non-Uniformity (GLN):

$$\boldsymbol{GLN} = \frac{\sum_{i=1}^{N_g}\left(\sum_{j=1}^{N_r} p(i,j|\theta)\right)^2}{N_z(\theta)}$$

4. Gray Level Non-Uniformity Normalized (GLNN):

$$\boldsymbol{GLNN} = \frac{\sum_{i=1}^{N_g}\left(\sum_{j=1}^{N_r} p(i,j|\theta)\right)^2}{N_z(\theta)^2}$$

5. Run Length Non-Uniformity (RLN):

$$\boldsymbol{RLN} = \frac{\sum_{j=1}^{N_r}\left(\sum_{i=1}^{N_g} P(i,j|\theta)\right)^2}{N_z(\theta)}$$

6. Run Length Non-Uniformity Normalized (RLNN):

$$\boldsymbol{RLNN} = \frac{\sum_{j=1}^{N_r}\left(\sum_{i=1}^{N_g} P(i,j|\theta)\right)^2}{N_z(\theta)^2}$$

RLNN measures the similarity of run lengths throughout the image, with a lower value indicating more homogeneity among run lengths in the image. This is the normalized version of the RLN formula.

7. Run Percentage (RP):

$$\boldsymbol{RP} = \sum_{i=1}^{N_g}\sum_{j=1}^{N_r}\frac{P(i,j|\theta)}{N_p}$$

8. Gray Level Variance (GLV):

$$\boldsymbol{GLV} = \sum_{i=1}^{N_g}\sum_{j=1}^{N_r} p(i,j|\theta)\,(i-\mu)^2$$

Here, $\boldsymbol{\mu} = \sum_{i=1}^{N_g}\sum_{j=1}^{N_r} p(i,j|\theta)\, i$

9. Run Variance (RV):

$$\boldsymbol{RV} = \sum_{i=1}^{N_g}\sum_{j=1}^{N_r} p(i,j|\theta)\,(j-\mu)^2$$

Here, $\boldsymbol{\mu} = \sum_{i=1}^{N_g}\sum_{j=1}^{N_r} p(i,j|\theta)\, j$

10. Run Entropy (RE):

$$RE = \sum_{i=1}^{N_g}\sum_{j=1}^{N_r} p(i,j|\theta)\log_2(p(i,j|\theta)+\epsilon)$$

Here, ϵ is an arbitrarily small positive number ($\approx 2.2\times 10^{-16}$)

11. Low Gray Level Run Emphasis (LGLRE):
LGLRE measures the distribution of low gray-level values, with a higher value indicating a greater concentration of low gray-level values in the image.

$$LGLRE = \frac{\sum_{i=1}^{N_g}\sum_{j=1}^{N_r}\frac{P(i,j|\theta)}{i^2}}{N_z(\theta)}$$

12. High Gray Level Run Emphasis (HGLRE):
HGLRE measures the distribution of the higher gray-level values, with a higher value indicating a greater concentration of high gray-level values in the image.

$$HGLRE = \frac{\sum_{i=1}^{N_g}\sum_{j=1}^{N_r} i^2 P(i,j|\theta)}{N_z(\theta)}$$

13. Short Run Low Gray Level Emphasis (SRLGLE):

$$SRLGLE = \frac{\sum_{i=1}^{N_g}\sum_{j=1}^{N_r}\frac{P(i,j|\theta)}{i^2 j^2}}{N_z(\theta)}$$

14. Short Run High Gray Level Emphasis (SRHGLE):

$$SRHGLE = \frac{\sum_{i=1}^{N_g}\sum_{j=1}^{N_r}\frac{P(i,j|\theta) i^2}{j^2}}{N_z(\theta)}$$

15. Long Run Low Gray Level Emphasis (LRLGLE):

$$LRLGLE = \frac{\sum_{i=1}^{N_g}\sum_{j=1}^{N_r}\frac{P(i,j|\theta) j^2}{i^2}}{N_z(\theta)}$$

16. Long Run High Gray Level Emphasis (LRHGLE):

$$LRHGLE = \frac{\sum_{i=1}^{N_g}\sum_{j=1}^{N_r} P(i,j|\theta) i^2 j^2}{N_z(\theta)}$$

Neigbouring Gray Tone Difference Matrix (NGTDM) Features
A NGTDM quantifies the difference between a gray value and the average gray value of its neighbours within distance δ. The sum of absolute differences for gray level i is stored in the matrix. Let X_{gl} be a set of segmented voxels and $x_{gl}(j_x, j_y, j_z) \in X_{gl}$ be the gray level of a voxel at postion (j_x, j_y, j_z), then the average gray level of the neigbourhood is:

$$\bar{A}_i = \bar{A}(j_x, j_y, j_z)$$

$$= \frac{1}{W}\sum_{k_x=-\delta}^{\delta}\sum_{k_y=-\delta}^{\delta}\sum_{k_z=-\delta}^{\delta} x_{gl}(j_x+k_x, j_y+k_y, j_z+k_z)$$

$$where\ (k_x, k_y, k_z) \neq (0,0,0) and\ x_{gl}(j_x+k_x, j_y+k_y, j_z+k_z) \in X_{gl}$$

Here, $\boldsymbol{W}$ is the number of voxels in the neighbourhood that are also in $\boldsymbol{X_{gl}}$.

1. Coarseness:
Coarseness is a measure of average difference between the center voxel and its neighbourhood. A higher value suggests a lower spatial change rate and a more uniform texture.

$$\boldsymbol{Coarseness = \frac{1}{\sum_{i=1}^{N_g} p_i\, s_i}}$$

2. Contrast:

$$\boldsymbol{Contrast = \left(\frac{1}{N_{g,p}\left(N_{g,p}-1\right)}\sum_{i=1}^{N_g}\sum_{j=1}^{N_g} p_i p_j\,(i-j)^2\right)\left(\frac{1}{N_{v,p}}\sum_{i=1}^{N_g} s_i\right), where\ \ p_i \neq 0, p_j \neq 0}$$

3. Busyness:

$$\boldsymbol{Busyness = \frac{\sum_{i=1}^{N_g} p_i s_i}{\sum_{i=1}^{N_g}\sum_{j=1}^{N_g} |ip_i - jp_j|}, where\ \ p_i \neq 0, p_j \neq 0}$$

4. Complexity:

$$\boldsymbol{Complexity = \frac{1}{N_{v,p}}\sum_{i=1}^{N_g}\sum_{j=1}^{N_g} |i-j|\ \frac{p_i s_i + p_j s_j}{p_i + p_j}, where\ \ p_i \neq 0, p_j \neq 0}$$

5. Strength:

$$\boldsymbol{Strength = \frac{\sum_{i=1}^{N_g}\sum_{j=1}^{N_g}\left(p_i + p_j\right)(i-j)^2}{\sum_{i=1}^{N_g} s_i}, where\ \ p_i \neq 0, p_j \neq 0}$$

Gray Level Dependence Matrix (GLDM) Features
A Gray Level Dependence Matrix (GLDM) quantifies gray level dependencies in an image. A gray level dependency is defined as the number of connected voxels within distance $\boldsymbol{\delta}$ that are dependent on the center voxel. A neighboring voxel with gray level $\boldsymbol{j}$ is considered dependent on center voxel with gray level $\boldsymbol{i}$ if $|\boldsymbol{i}-\boldsymbol{j}| \leq \boldsymbol{\alpha}$. In a gray level dependence matrix $\boldsymbol{P(i,j)}$ the $(\boldsymbol{i},\boldsymbol{j})^{\mathbf{th}}$ element describes the number of times a voxel with gray level $\boldsymbol{i}$ with $\boldsymbol{j}$ dependent voxels in its neighborhood appears in image.

Let:
- $\boldsymbol{N_g}$ be the number of discreet intensity values in the image,
- $\boldsymbol{N_d}$ be the number of discreet dependency sizes in the image,
- $\boldsymbol{N_z}$ be the number of dependency zones in the image, which is equal to $\boldsymbol{\sum_{i=1}^{N_g}\sum_{j=1}^{N_d} P(i,j)}$
- $\boldsymbol{P(i,j)}$ be the dependence matrix,
- $\boldsymbol{p(i,j)}$ be the normalized dependence matrix, defined as $\boldsymbol{p(i,j) = \frac{P(i,j)}{N_z}}$.

1. Small Dependence Emphasis (SDE):

$$\boldsymbol{SDE = \frac{\sum_{i=1}^{N_g}\sum_{j=1}^{N_d}\frac{P(i,j)}{i^2}}{N_z}}$$

2. Large Dependence Emphasis (LDE):

$$\boldsymbol{LDE} = \frac{\sum_{i=1}^{N_g} \sum_{j=1}^{N_d} \boldsymbol{P(i,j) j^2}}{\boldsymbol{N_z}}$$

3. Gray Level Non-Uniformity (GLN):

$$\boldsymbol{GLN} = \frac{\sum_{i=1}^{N_g} (\sum_{j=1}^{N_d} \boldsymbol{P(i,j)})^2}{\boldsymbol{N_z}}$$

4. Dependence Non-Uniformity (DN):

$$\boldsymbol{DN} = \frac{\sum_{j=1}^{N_d} (\sum_{i=1}^{N_g} \boldsymbol{P(i,j)})^2}{\boldsymbol{N_z}}$$

5. Dependence Non-Uniformity Normalized (DNN):

$$\boldsymbol{DN} = \frac{\sum_{j=1}^{N_d} (\sum_{i=1}^{N_g} \boldsymbol{P(i,j)})^2}{{N_z}^2}$$

6. Gray Level Variance (GLV):

$$GLV = \sum_{i=1}^{N_g} \sum_{j=1}^{N_d} p(i,j)\,(i-\mu)^2, where\ \mu = \sum_{i=1}^{N_g} \sum_{j=1}^{N_d} ip(i,j)$$

7. Dependence Variance (DV):

$$DV = \sum_{i=1}^{N_g} \sum_{j=1}^{N_d} p(i,j)\,(j-\mu)^2, where\ \mu = \sum_{i=1}^{N_g} \sum_{j=1}^{N_d} jp(i,j)$$

8. Dependence Entropy (DE):

$$Dependence\ Entropy = -\sum_{i=1}^{N_g} \sum_{j=1}^{N_d} p(i,j) \log_2[p(i,j) + \epsilon]$$

9. Low Gray Level Emphasis (LGLE):

$$LGLE = \frac{\sum_{i=1}^{N_g} \sum_{j=1}^{N_d} \frac{P(i,j)}{i^2}}{N_z}$$

10. High Gray Level Emphasis (HGLE):

$$HGLE = \frac{\sum_{i=1}^{N_g} \sum_{j=1}^{N_d} P(i,j) i^2}{N_z}$$

11. Small Dependence Low Gray Level Emphasis (SDLGLE):

$$SDLGLE = \frac{\sum_{i=1}^{N_g} \sum_{j=1}^{N_d} \frac{P(i,j)}{i^2 j^2}}{N_z}$$

12. Small Dependence High Gray Level Emphasis (SDHGLE):

Measures the joint distribution of small dependence with higher gray-level values.

13. Large Dependence Low Gray Level Emphasis (LDLGLE):

$$LDLGLE = \frac{\sum_{i=1}^{N_g} \sum_{j=1}^{N_d} \frac{P(i,j) i^2}{j^2}}{N_z}$$

14. Large Dependence High Gray Level Emphasis (LDHGLE):

$$LDHGLE = \frac{\sum_{i=1}^{N_g} \sum_{j=1}^{N_d} P(i,j) i^2 j^2}{N_z}$$

Table S1. Linear regression of the percent change in lymph node volume and each radiomic feature.

Feature	Estimate	Standard Error	Adjusted p-value
Mean	-0.001626031	0.000541	0.007269
Minimum	0.004498638	0.000943	9.49E-05
Maximum	-5.77E-04	0.000102	1.06E-05
GrayLevelVariance1	-3.14E-03	0.000659	9.54E-05
HighGrayLevelEmphasis	-0.001100407	0.000235	0.000111
DependenceEntropy	-0.159587788	0.045063	0.001935
DependenceNonUniformity	-0.000216466	0.000155	0.198385
GrayLevelNonUniformity1	-4.38E-05	0.000112	0.733756
SmallDependenceEmphasis	-2.15E+00	0.428914	5.30E-05
SmallDependenceHighGrayLevelEmphasis	-0.001587803	0.00035	0.000151
DependenceNonUniformityNormalized	-6.82E-01	0.80909	0.439257
LargeDependenceEmphasis	0.013084829	0.004218	0.005747
LargeDependenceLowGrayLevelEmphasis	0.006766132	0.005021	0.215221
DependenceVariance	-0.097848561	0.076109	0.235716
LargeDependenceHighGrayLevelEmphasis	-0.000210783	6.59E-05	0.004581
SmallDependenceLowGrayLevelEmphasis	16.82738351	10.43382	0.138923
LowGrayLevelEmphasis	5.15E-01	3.28E-01	0.148632
JointAverage	-4.52E-02	0.009132	6.05E-05
SumAverage	-2.26E-02	0.004566	6.05E-05
JointEntropy	-0.135407497	0.027002	5.21E-05
ClusterShade	-1.20E-04	1.88E-05	1.72E-06
MaximumProbability	0.495796193	0.270476	0.091754
Idmn	-17.89280799	15.33538	0.280269
JointEnergy	0.549536722	0.346648	0.14521

Contrast1	-0.024920315	0.005575	0.00018
DifferenceEntropy	-0.363917398	0.071111	4.20E-05
InverseVariance	-0.378971851	0.749883	0.65982
DifferenceVariance	-0.051960633	0.011362	0.000139
Idn	-1.232120613	4.348089	0.813988
Idm	1.74548333	0.361679	8.38E-05
Correlation	-0.777068449	0.349495	0.042135
Autocorrelation	-1.10E-03	2.36E-04	0.000118
SumEntropy	-0.173798618	0.037402	0.000118
MCC	-0.855961669	0.383781	0.041779
SumSquares	-0.003147062	0.000667	0.000104
ClusterProminence	-4.26E-07	8.43E-08	4.96E-05
Imc2	-0.796440756	0.378012	0.053054
Imc1	0.484907103	0.441603	0.309532
DifferenceAverage	-0.253301842	0.053245	9.54E-05
Id	1.936287393	0.411745	0.000106
ClusterTendency	-0.00081232	0.000172	0.000102
InterquartileRange	-1.22E-03	2.68E-04	0.000141
Skewness	-0.082707743	0.031859	0.018921
Uniformity	0.729825084	3.10E-01	0.032624
Median	-1.52E-03	5.46E-04	0.01222
Energy	-2.16E-09	4.03E-10	2.34E-05
RobustMeanAbsoluteDeviation	-0.002932395	0.000638	0.000134
MeanAbsoluteDeviation	-2.60E-03	0.000518	5.15E-05
TotalEnergy	-3.02E-09	5.67E-10	2.51E-05
RootMeanSquared	-1.46E-03	0.000366	0.00061
x90Percentile	-0.000866478	0.000212	0.000465
Entropy	-0.207291693	0.041223	5.15E-05
Range	-5.55E-04	9.13E-05	3.48E-06
Variance	-5.03E-06	1.06E-06	9.54E-05
x10Percentile	0.004493115	0.001061	0.000325
Kurtosis	-	0.008678	0.309532

	0.009500933		
ShortRunLowGrayLevelEmphasis	2.672487376	1.346469	0.068011
GrayLevelVariance2	-3.53E-03	0.000684	3.73E-05
LowGrayLevelRunEmphasis	1.203728907	0.524148	0.03662
GrayLevelNonUniformityNormalized	2.116888673	0.418427	4.88E-05
RunVariance	-0.000248706	1.86E-03	0.919434
GrayLevelNonUniformity2	-0.000326381	0.00082	0.733217
LongRunEmphasis	1.41E-04	0.001423	0.936974
ShortRunHighGrayLevelEmphasis	-0.000986611	0.000206	9.14E-05
RunLengthNonUniformity	-9.86E-04	0.000178	1.44E-05
ShortRunEmphasis	-1.740249675	0.339448	4.15E-05
LongRunHighGrayLevelEmphasis	-9.30E-05	7.33E-05	0.240837
RunPercentage	-1.319564038	0.346924	0.000985
LongRunLowGrayLevelEmphasis	0.004808328	5.31E-03	0.405437
RunEntropy	-3.34E-01	0.070613	0.000101
HighGrayLevelRunEmphasis	-0.000943538	0.000196	8.41E-05
RunLengthNonUniformityNormalized	-1.757288882	0.325389	2.08E-05
GrayLevelVariance3	-4.39E-03	6.90E-04	1.82E-06
ZoneVariance	-2.56E-07	2.03E-06	0.920288
GrayLevelNonUniformityNormalized.1	1.44E+00	0.410148	0.00214
SizeZoneNonUniformityNormalized	-1.497610202	3.50E-01	0.000294
SizeZoneNonUniformity	-2.66E-03	0.000436	3.40E-06
GrayLevelNonUniformity3	-1.35E-02	0.00621	0.047321
LargeAreaEmphasis	-9.51E-08	1.97E-06	0.961824
SmallAreaHighGrayLevelEmphasis	-1.01E-03	1.85E-04	1.70E-05
ZonePercentage	-1.94E+00	0.385564	5.15E-05
LargeAreaLowGrayLevelEmphasis	1.11E-05	1.54E-05	0.51011
LargeAreaHighGrayLevelEmphasis	7.27E-09	1.17E-07	0.958121
HighGrayLevelZoneEmphasis	-8.59E-04	0.000155	1.51E-05
SmallAreaEmphasis	-1.026289476	0.288512	0.001871
LowGrayLevelZoneEmphasis	8.68E-01	4.03E-01	0.048506
ZoneEntropy	-0.190744089	4.33E-02	0.000212
SmallAreaLowGrayLevelEmphasis	0.923948182	0.710611	0.231511
Coarseness	-0.978376516	4.979076	0.873828
Complexity	-5.64E-04	9.06E-05	2.55E-06

Strength	-1.67E-02	0.00267	2.35E-06
Contrast2	-3.56506159	0.915698	0.000788
Busyness	0.022161528	0.015721	0.196002

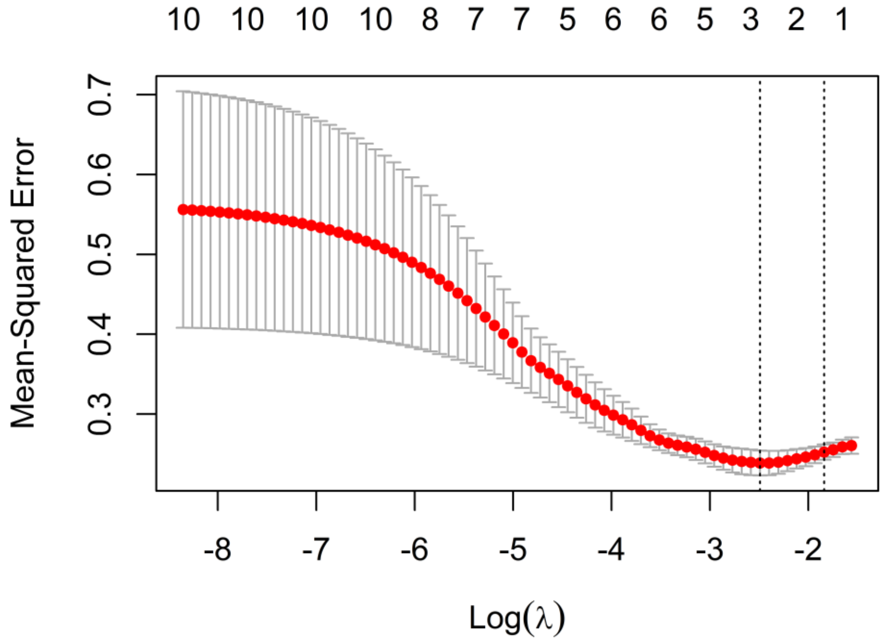

Figure S1. λ optimization by 5-fold LASSO regression. The horizontal axis at the top represents the number of non-zero features in the model as λ is adjusted. The optimal λ minimizing mean-squared error is plotted as the leftward vertical dotted line, with 3 non-zero features in our model.
